# A case of diagnosis and treatment of a patient with penetrating injury of the carotid artery: Case report

**DOI:** 10.1097/MD.0000000000042364

**Published:** 2025-05-16

**Authors:** Shaou Wang, Canyun Wang, Yonggang Qiu, Jiajia Lu, Hao Dong

**Affiliations:** a Department of Ultrasound, The First People’s Hospital of Xiaoshan District, Xiaoshan Affiliated Hospital of Wenzhou Medical University, Hangzhou, Zhejiang, China; b Department of Radiology, The First People’s Hospital of Xiaoshan District, Xiaoshan Affiliated Hospital of Wenzhou Medical University, Hangzhou, Zhejiang, China.

**Keywords:** carotid artery, computed tomography angiography, neck trauma, open injury, penetrating wound

## Abstract

**Rationale:**

Carotid artery penetrating injury is rare, but the disability and mortality rates are high. Timely and correct diagnosis, selection of reasonable surgical access and appropriate vascular repair method can shorten the operation time and reduce the incidence of postoperative cerebral ischemic-hypoxic complications.

**Patient concerns:**

We report a 22-year-old man who stabbed himself in the right neck with a knife after drinking and was admitted to our hospital.

**Diagnoses:**

Preoperatively, we use bedside ultrasound and computed tomography angiography to accurately determine the location and size of the wound.

**Interventions:**

After diagnosing the patient with a carotid artery penetrating injury, we promptly performed a repair of his carotid artery penetrating injury, taking into account the location and size of the wound.

**Outcomes:**

The patient recovered well after surgery and was reviewed half a month later without neurologic complications.

**Lessons:**

The successful resuscitation of this case is a good inspiration for our primary care physicians, and it is worthwhile to learn from the selection of examination methods and principles of treatment when facing this kind of patients with neck trauma in the future.

## 1. Introduction

The neck is a vulnerable site to injury due to the absence of skeletal protection. The neck contains vital structures like the trachea, esophagus, blood arteries, and nervous system structures. Neck injuries provide a significant risk of life-threatening conditions, strongly associated with the anatomical arrangement of vital structures in the neck and their interconnections.^[1,2]^ Carotid artery injury accounts for 6% to 13% of all penetrating neck injury and up to 22% of neck vascular injuries.^[3,4]^ The common carotid artery is the most often injured artery (73%), followed by the middle cerebral artery (22%) and the subclavian artery (5%).^[3]^ The predominant mode of injury is gunshot wounds, succeeded by stab wounds, and subsequently injuries from pieces or shrapnel.^[5,6]^

## 2. Case presentation

A 22-year-old male patient suffered a stab wound to the right neck from a fruit knife after consuming alcohol 3 hours before, resulting in significant hemorrhage and pain in the afflicted region. Following the self-removal of the fruit knife, the patient received assistance from family and friends for compression hemostasis and was then transported to the emergency department of our hospital by the 120 ambulance service. Upon admission, the patient exhibited clear consciousness, appropriate responses to inquiries, and poor mood. There were no signs of nausea, vomiting, chest tightness, dyspnea, chest pain, palpitations, or coma, and he denied any history of additional injuries. In the physical examination, he was conscious and his vital signs exhibited a pulse of 88 beats per min, the respiration of 15 times/min, the blood pressure of 120/75 mm Hg, and the body temperature of 37°C. A specialist examination identified a 1 cm oblique and regular incision in the central region of the right neck, accompanied by obvious swelling and tenderness in the surrounding tissues. The trachea deviated to the left, whereas the remainder of the physical examination revealed no abnormalities. The patient reported a history of depression exceeding 1 year and has been administered sertraline and lorazepam orally for a long time. The medication was withdrawn by himself for more than 4 months. He denied the history of other diseases and drug allergy.

**Figure 1.**
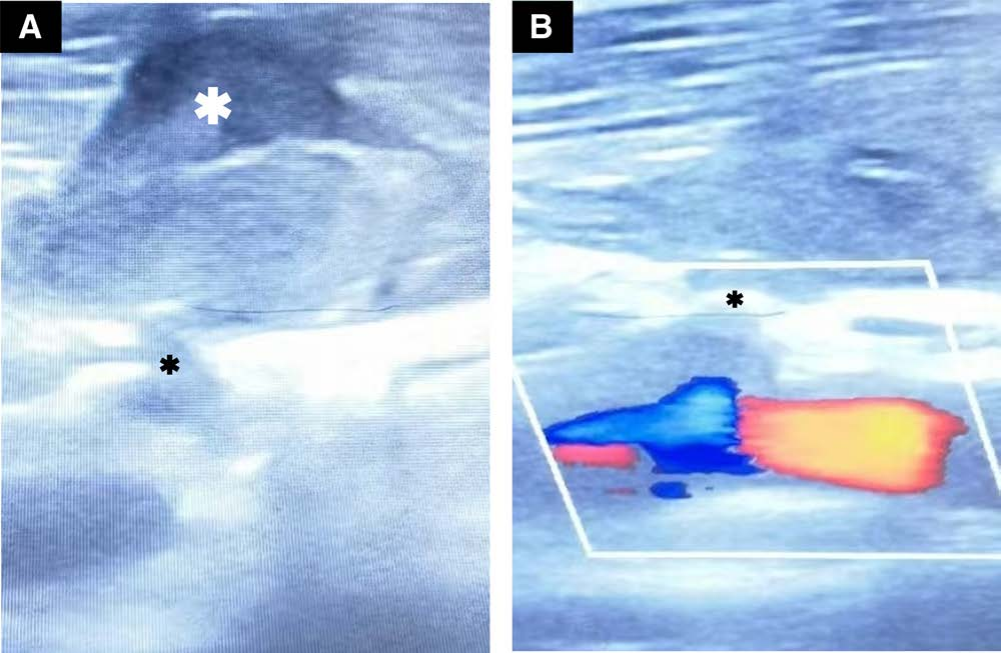
Bedside ultrasound images. (A) The rupture at the right common carotid artery bifurcation (shown by black asterisk) and the hematoma immediately above it (shown by white asterisk). (B) Color flow status showing the breach (shown by black asterisks).

**Figure 2.**
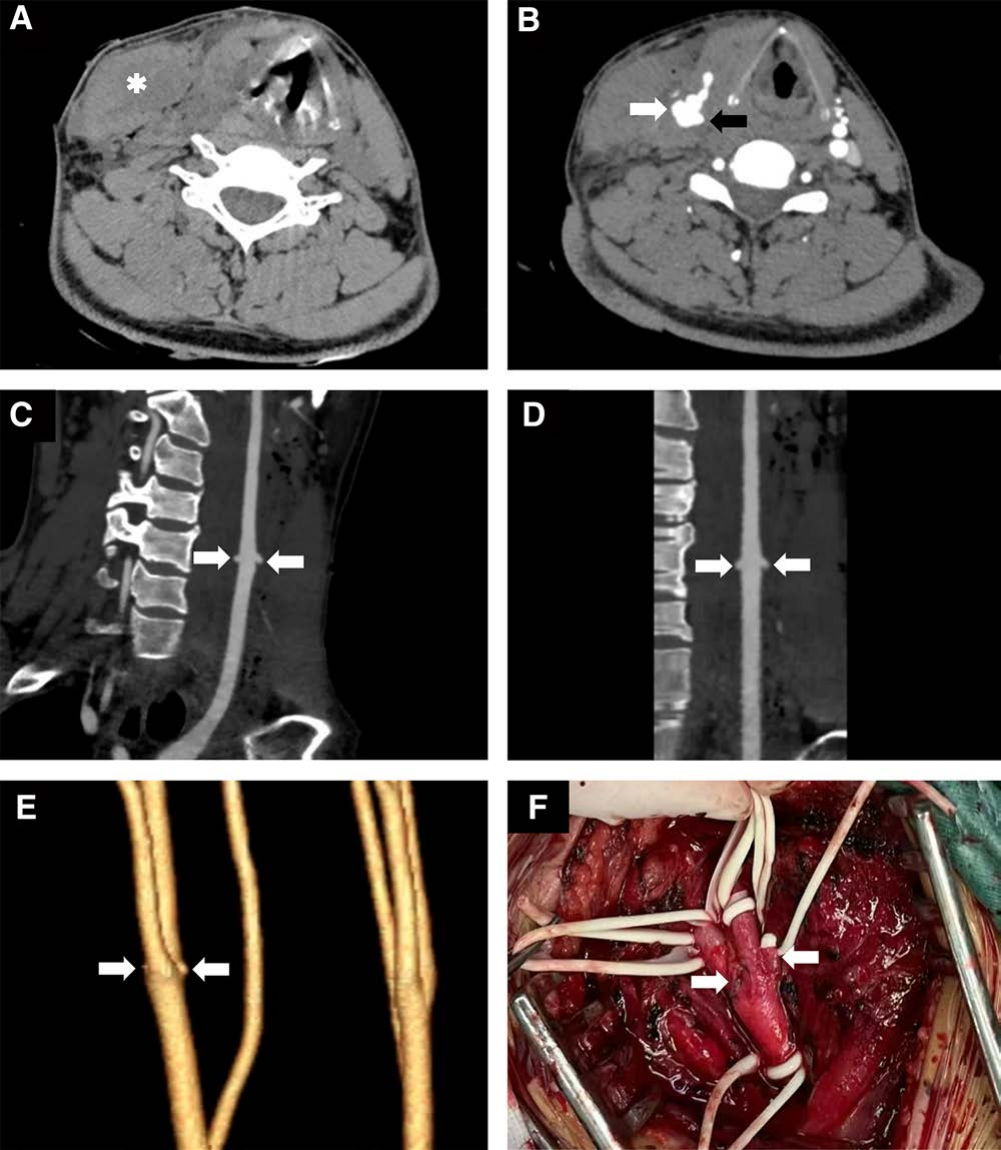
CTA and surgical images. (A) CT plain axial view showing a right anterior carotid hematoma (white asterisk). (B) CT arterial enhancement (CTA) in axial position shows the external (white arrows) and internal (black arrows) portions of the carotid penetrating injury rupture. (C, D) CTA reconstruction images clearly show the carotid artery penetrating injury breach (white arrow). (E) VR image showing the penetrating wound rupture at the bifurcation of the common carotid artery (white arrow). (F) Intraoperative demonstration of a sutured penetrating wound breach at the bifurcation of the common carotid artery (white arrow). CT = computed tomography, CTA = computed tomography angiography, VR = volume rendering.

Upon admission, bedside emergency ultrasonography of the carotid artery and bilateral internal jugular veins revealed a very modest echogenicity of approximately 2.6 × 1.9 cm located above the bifurcation of the right common carotid artery. The boundary was discernible. A duct around 3 mm in width was connected to the common carotid artery. Narrow blood flow signals could be detected in the duct (Fig. 1). The right internal jugular vein could not be clearly examined. Ultrasonography illustrated that hematoma above the bifurcation of the right common carotid artery was considered as pseudoaneurysm requiring evacuation. The thyroid ultrasonography revealed a hypoechoic region of 3.6 × 1.6 cm adjacent to the right lobe of the thyroid gland, characterized by a distinct boundary, an absence of significant blood flow signals and thyroid gland appeared normal. Ultrasonography also suggested that right thyroid lobe hematoma was possible. To further clarify the carotid artery injury of the patient, a repeat carotid artery computed tomography angiography (CTA) examination was conducted. The CTA revealed 2 prominent alterations near the bifurcation of the right common carotid artery, accompanied by patchy high-density shadows in the surrounding area. The rest bilateral carotid arteries and vertebral arteries followed a normal trajectory. It was diagnosed that the patient was suspected penetrating injury at the bifurcation of the right common carotid artery accompanied by adjacent hematoma development (Fig. 2A–E).The results of the chest computed tomography, cranial computed tomography, and electrocardiogram were unremarkable.

The patient is in emergency surgery. Intraoperatively, a significant right-sided cervical hematoma of about 3 cm was seen. Visible hematoma within the right sternocleidomastoid muscle, a wound of approximately 3 mm was seen on the anterior wall of the right internal carotid artery at its origin and on the posterior wall of the right common carotid artery at its bifurcation, localized blood clot formation. Removal of the clot was followed by a jet of hemorrhage with a jugular vein wound of about 2 mm at its corresponding site. After systemic heparinization (4000 U), the blood pressure was raised to 150 mm Hg, and the internal carotid, superior thyroid, external carotid and common carotid arteries were successively blocked to control the hemorrhage, then 2 wounds of the carotid arteries were closed with 6-0 prolene continuous sutures (Fig. 2F), and after the closure of the sutures, the superior thyroid, external carotid, and common carotid arteries were opened sequentially. The internal carotid artery was opened after blood drainage from the suture incision, and no active bleeding was seen, and the internal carotid artery and external carotid artery were well pulsed. The carotid arteries were blocked for a total of 18 minutes, and the blood pressure was controlled and stabilized. The jugular vein wound was closed with 5-0 prolene suture, checking for no bleeding, careful debridement, gelatin sponge piece to reduce exudation, the wound was placed with drainage tube and then sutured layer by layer, intraoperative anesthesia was smooth, the bleeding was 800 mL, and the autologous blood was transfused back to the patient for 650 mL. The process was smooth. Postoperatively, the patient’s vital signs were stable. Ventilator-assisted mechanical ventilation via orotracheal intubation. Enoxaparin sodium injection of 4000 AxaIU was then administered subcutaneously quaque die. The patient was then transferred to intensive care unit for observation, and was given cefuroxime injection to prevent postoperative wound infection and symptomatic treatments such as sputum, gastric protection and rehydration, etc. The patient was extubated after 1 day, and respiratory oxygenation was maintained. On the 2nd postoperative day, the patient was transferred from the intensive care unit to the thoracic surgery department for further treatments. After 3 days, the patient was discharged with medication as the neck incision was healing well without significant erythema and oozing. Half a month later, the patient returned to our hospital for review as prescribed, and recovered well with no significant complications.

## 3. Discussion

Penetrating neck injury can be categorized into 3 zones based on the anatomical level of injury. Zone I spans from the clavicle and sternal notch to the cricoid cartilage level. Zone II encompasses the region between the cricoid cartilage and the inferior margin of the mandible. Zone III is the area between the mandibular angle and the skull base.^[7]^ Monson’s classification of neck zones indicates that the carotid artery has the largest traversable path in zone II and is particularly susceptible to injury due to the absence of bone protection.^[8]^ This is corroborated by the occurrence of the injury in zone II in this case.

Penetrating injuries to the carotid artery are rather uncommon. They present a therapeutic challenge for surgeons due to their tendency for active hemorrhage, the development of expanding hematomas that may obstruct the airway, and the risk of severe neurological sequelae linked to elevated disability and fatality rates.^[4]^ Therefore, prompt and precise preoperative radiological assessment and the exclusion of injuries to critical neck structures are essential. Preoperative assessments comprised doppler ultrasonography, CTA, magnetic resonance angiography, and digital subtraction angiography. The benefit of digital subtraction angiography is its ability to provide details regarding the location, severity, and extent of vascular injury. It can also execute therapeutic procedures for hemorrhage control, embolization, stent placement, or temporary balloon closure of the afflicted artery to assess the potential neurological effects of ligation. Nevertheless, it remains an invasive procedure that may result in serious complications, including hematoma, pseudoaneurysm at the puncture site, thrombosis, detachment of distal atherosclerotic plaques, and arterial dissection. These complications can lead to catastrophic neurological sequelae.^[9]^ CTA is a noninvasive, rapid, and effective alternative to arteriography, capable of accurately identifying clinically significant vascular injuries in the neck while also providing insights into related soft tissue injuries, cervical vertebrae, and the airway digestive tract, thereby minimizing unnecessary surgical exploration. In comparison to traditional angiography, CTA exhibits superior sensitivity and specificity in diagnosing carotid artery injury.^[9]^ Consequently, neck CTA may serve as a preliminary approach for patients with carotid artery injuries.^[10]^ Doppler is a prevalent diagnostic imaging technique for assessing neck zone II injuries, attributed to its affordability, absence of radiation, and effective diagnostic capabilities.^[3,11]^ However, the results are contingent upon the operator, and vascular injuries in neck zones I and III may be overlooked, particularly in obese patients or in situations involving extensive hemorrhage or emphysema.^[12]^ Magnetic resonance angiography is impractical for trauma patients due to its inadequate evaluation of concomitant bone injuries, prolonged duration, and incompatibility with implants and metallic devices.^[13]^ In this case, preoperative ultrasonography and CTA assessments were integrated to ascertain the location, size, and adjacent anatomical structures of the patient’s carotid artery injury, thereby informing doctors in selecting a suitable surgical strategy and vascular repair technique.

As surgical techniques advanced, carotid artery repair become as the preferred treatment when feasible.^[14-16]^ Repair of an injured carotid artery, when performed without neurological impairment, results in lower disability and fatality rates compared to straightforward vascular ligation. Repair techniques often encompass straightforward suturing of the vessel wall, end-to-end anastomosis, and transplantation of artificial blood vessels and so on.^[13]^ The lateral artery may be directly sutured for mild injury. Secondly, for serious injuries, end-to-end anastomosis must be executed, ensuring that no tension is present at the anastomosis site. Ultimately, for significant, destructive lesions, autologous or synthetic blood vessels may be utilized for anastomosis.^[3]^ Furthermore, some experts have suggested that nonsurgical interventions may be appropriate for select patients.^[17]^ In this case, a 3-mm wound was seen on the anterior wall of the internal carotid artery and the posterior wall of the bifurcation of the common carotid artery. Fortunately, both injuries were obstructed by hematomas. The hemodynamics were temporarily stable, and no neurological complications. The physician conducted vascular repair on the patient, who subsequently experienced a favorable recovery postoperation.

In summary, prompt diagnosis, ensuring a patent airway, selecting an appropriate surgical technique and vascular repair method, and promptly restoring carotid blood flow are essential for minimizing mortality rates and neurological complications associated with open carotid artery injury.

## Author contributions

**Conceptualization:** Shaou Wang, Jiajia Lu.

**Data curation:** Shaou Wang, Canyun Wang, Hao Dong.

**Project administration:** Yonggang Qiu.

**Writing – original draft:** Shaou Wang.

**Writing – review & editing:** Hao Dong.
